# Health Care Provider Counseling for Physical Activity or Exercise Among Adults with Arthritis — United States, 2002 and 2014

**DOI:** 10.15585/mmwr.mm665152a2

**Published:** 2018-01-05

**Authors:** Jennifer M. Hootman, Louise B. Murphy, John D. Omura, Teresa J. Brady, Michael Boring, Kamil E. Barbour, Charles G. Helmick

**Affiliations:** ^1^Division of Population Health, National Center for Chronic Disease Prevention and Health Promotion, CDC; ^2^Division of Nutrition, Physical Activity and Obesity, National Center for Chronic Disease Prevention and Health Promotion, CDC.

Arthritis affects an estimated 54 million U.S. adults and, as a common comorbidity, can contribute arthritis-specific limitations or barriers to physical activity or exercise for persons with diabetes, heart disease, and obesity (*1*). The American College of Rheumatology’s osteoarthritis management guidelines recommend exercise as a first-line, nonpharmacologic strategy to manage arthritis symptoms (*2*), and a *Healthy People 2020* objective is to increase health care provider counseling for physical activity or exercise among adults with arthritis.* To determine the prevalence and percentage change from 2002 to 2014 in receipt of health care provider counseling for physical activity or exercise (counseling for exercise) among adults with arthritis, CDC analyzed 2002 and 2014 National Health Interview Survey (NHIS) data. From 2002 to 2014, the age-adjusted prevalence of reporting health care provider counseling for exercise among adults with arthritis increased 17.6%, from 51.9% (95% confidence interval [CI] = 49.9%–53.8%) to 61.0% (CI = 58.6%–63.4%) (p<0.001). The age-adjusted prevalence of reporting health care provider counseling for exercise among persons with arthritis who described themselves as inactive increased 20.1%, from 47.2% (CI = 44.0%–50.4%) in 2002 to 56.7% (CI = 52.3%–61.0%) in 2014 (p = 0.001). Prevalence of counseling for exercise has increased significantly since 2002; however, approximately 40% of adults with arthritis are still not receiving counseling for exercise. Improving health care provider training and expertise in exercise counseling and incorporating prompts into electronic medical records are potential strategies to facilitate counseling for exercise that can help adults manage their arthritis and comorbid conditions.

NHIS is an ongoing survey of the civilian, noninstitutionalized U.S. population that gathers data on a variety of health topics. CDC analyzed data from 2002 (adult respondents aged ≥18 years = 31,044; response rate = 74.3%) and 2014 (36,697 adults; response rate = 58.9%).^†^ Arthritis was defined as a “yes” response to the question, “Have you ever been told by a doctor or other health care professional that you have arthritis, rheumatoid arthritis, gout, lupus or fibromyalgia?” Health care provider counseling for exercise was defined as a “yes” response to the question “Has a doctor or other health professional ever suggested physical activity or exercise to help your arthritis or joint symptoms?” Age-adjusted percentages and CIs for health care provider counseling for exercise were calculated overall and by sociodemographic and health-related characteristics. Physical activity was calculated as minutes per week of moderate-intensity physical activity using six questions regarding the (typical/usual) frequency, intensity, and duration of aerobic physical activity. The level was categorized as active (≥150 minutes/week moderate-intensity equivalent activity), insufficiently active (some moderate-intensity equivalent activity but not enough to meet active definition), and inactive (no moderate-intensity equivalent activity that lasted at least 10 minutes). Age-adjusted prevalence ratios (PRs) to assess the relationship between counseling for exercise and physical inactivity were calculated using logistic regression.

Changes in age-adjusted prevalence of counseling for exercise were examined across the 5 years (2002, 2003, 2006, 2009, and 2014) in which both arthritis and counseling for exercise questions were included on the survey. All analyses included adjustment for the multistage complex survey design, including applying sampling weights to make estimates representative of the U.S. civilian, noninstitutionalized population. Estimates were age-standardized to the 2000 projected U.S. population using three age groups (18–44 years, 45–64 years, and ≥65 years).^§^ Statistically significant differences (p<0.05) in percentages were determined using t-tests.

In 2002 and 2014, the age-adjusted prevalences of health care provider counseling for exercise among adults with arthritis were 51.9% and 61.0%, respectively, representing a 17.6% increase (p<0.001) (Figure). In 2014, all subgroups exceeded the *Healthy People 2020* age-standardized target of 57.4% for adults with arthritis, with the exception of non-Hispanic other races (53.8%), underweight/normal weight persons (50.0%), current smokers (56.9%), inactive persons (56.7%), and persons without a primary care provider (50.7%). In 2002 and 2014, age-adjusted prevalences of health care provider counseling for exercise among adults with arthritis who were inactive were 47.2% and 56.7%, respectively, representing a 20.1% increase (p = 0.001) (Table). Overall, adults with arthritis and obesity had a higher prevalence of having received counseling for exercise than did those who were underweight/normal weight (70.7% versus 50.0% in 2014), but prevalence estimates by activity status were not statistically different within body mass index categories.

**FIGURE:**
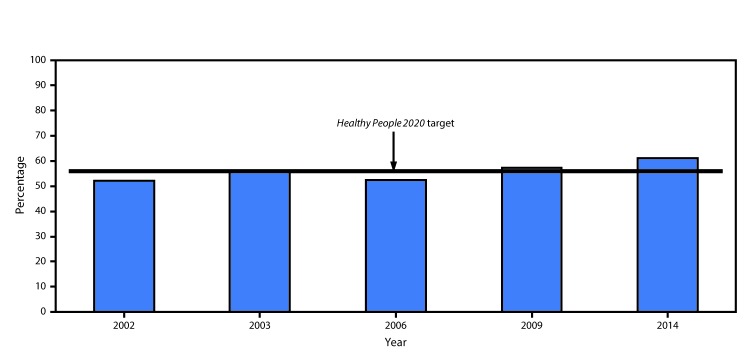
Percentage of adults with arthritis who reported receiving health care provider counseling for exercise — National Health Interview Survey, United States, 2002, 2003, 2006, 2009, and 2014

**TABLE:** Percentage of adults with arthritis who reported receiving health care provider counseling for exercise, by selected characteristics — National Health Interview Survey, United States, 2002 and 2014

Characteristic	2002				2014				% change 2002 to 2014^¶^
	No. in sample*	No. in U.S.^†^ (thousands)	Unadjusted % (95% CI)	Age-adjusted^§^ % (95% CI)	No. in sample*	No. in U.S.^†^ (thousands)	Unadjusted % (95% CI)	Age-adjusted^§^ % (95% CI)	
**Overall**	**3,572**	**22,355**	**52.8 (51.3–54.3)**	**51.9 (49.9–53.8)**	**5,639**	**33,108**	**61.6 (60.2–63.1)**	**61.0 (58.6–63.4)**	**17.6**
**Age group (yrs)**									
18–44	616	4,214	50.1 (46.8–53.4**)**	50.1 (46.8–53.4**)**	693	4,750	59.9 **(**55.7–64.0**)**	59.9 **(**55.7–64.0**)**	19.6
45–64	1,545	10,220	55.6 (53.4–57.8**)**	55.6 (53.4–57.8**)**	2,340	15,184	63.4 **(**61.2–65.5**)**	63.4 **(**61.2–65.5**)**	14.0
≥65	1,411	7,921	50.9 (48.8–53.0**)**	50.9 (48.8–53.0**)**	2,606	13,174	60.4 **(**58.3–62.4**)**	60.4 **(**58.3–62.4**)**	18.6
**Sex**									
Male	1,084	7,815	46.7 (44.5–49.0**)**	44.8 (42.0–47.7**)**	1,910	12,683	58.7 **(**56.4–61.1**)**	58.3 **(**54.7–61.9**)**	30.2
Female	2,488	14,540	56.7 (55.0–58.5**)**	56.8 (54.4–59.2**)**	3,729	20,425	63.6 **(**61.9–65.3**)**	62.9 **(**59.8–66.0)	10.8
**Race/Ethnicity**									
White, non-Hispanic	2,619	17,867	52.1 (50.4–53.7**)**	51.1 (48.8–53.3**)**	3,909	24,838	60.5 **(**58.8–62.2**)**	60.9 **(**57.9–63.8)	19.2
Black, non-Hispanic	530	2,636	58.5 (54.8–62.2**)**	59.0 (54.1–63.8**)**	894	4,022	64.9 **(**61.4–68.3**)**	63.0 **(**57.6–68.1)	6.7
Hispanic	362	1,412	53.8 (49.1–58.3**)**	52.3 (47.2–57.4**)**	597	3,120	67.5 **(**63.0–71.6**)**	64.7 **(**58.6–70.4)	23.7
Other race, non-Hispanic	61	440	48.4 (38.3–58.6**)**	43.4 (33.3–54.0**)**	239	1,127	61.0 **(**52.2–69.2**)**	53.8 **(**41.3–65.8)	24.1
**Education**									
Less than high school graduate	739	3,896	45.9 (43.0–48.8**)**	45.9 (41.2–50.7**)**	988	4,998	59.0 **(**55.6–62.3**)**	59.0 **(**52.6–65.0)	28.5
High school graduate or equivalent	1,087	7,137	52.3 (49.7–54.9**)**	49.8 (46.2–53.4**)**	1,554	9,204	59.9 **(**56.9–62.9**)**	58.1 **(**53.5–62.5)	16.7
Technical school/Some college	1,039	6,541	56.3 (53.8–58.8**)**	55.2 (52.2–58.1**)**	1,730	10,379	62.9 **(**60.5–65.4**)**	64.2 **(**60.6–67.6)	16.4
University degree	680	4,614	56.0 (52.8–59.2**)**	55.1 (50.9–59.2**)**	1,346	8,362	63.6 **(**60.8–66.4**)**	60.9 **(**56.0–65.6)	10.6
**Work status**									
Employed	1,430	9,899	52.5 (50.3–54.7**)**	51.2 (48.8–53.7**)**	2,042	13,518	61.1 **(**58.7–63.5**)**	60.4 **(**57.2–63.5)	18.0
Unemployed	86	484	44.6 (36.4–53.1**)**	47.0 (38.2–55.9**)**	205	1,381	62.7 **(**55.0–69.7**)**	61.0 **(**52.3–69.0)	29.8
Unable to work/ Disabled	588	3,244	54.8 (51.3–58.2)	51.4 (46.4–56.3)	1,024	5,312	64.6 (61.3–67.8)	63.9 (58.5–69.0)	24.3
Other	1,464	8,710	53.0 (50.9–55.0**)**	59.8 (54.1–65.3**)**	2,365	12,890	60.9 **(**58.8–63.0**)**	58.7 **(**51.3–65.8)	−1.8
**Arthritis limitations**									
Limited by arthritis	1,626	9,563	60.2 (58.1–62.3**)**	58.4 (55.3–61.4**)**	2,696	15,253	67.7 **(**65.4–69.9**)**	65.7 **(**61.4–69.8)	12.6
Not limited by arthritis	1,940	12,762	48.3 (46.3–50.2**)**	48.1 (45.7–50.6**)**	2,939	17,826	57.3 **(**55.4–59.1**)**	57.8 **(**54.9–60.6)	20.0
**Self-rated health**									
Excellent/Very good	1,196	7,945	49.2 (46.8–51.5**)**	49.0 (46.2–51.8**)**	1,939	12,350	58.7 **(**56.6–60.8**)**	58.4 **(**55.1–61.7)	19.2
Good	1,203	7,759	55.4 (53.0–57.7**)**	54.3 (50.9–57.6**)**	1,929	11,353	63.9 **(**61.2–66.6**)**	61.8 **(**57.0–66.4)	14.0
Fair/Poor	1,170	6,637	54.8 (52.3–57.3**)**	53.9 (50.0–57.7**)**	1,770	9,400	63.0 **(**60.5–65.5**)**	64.6 **(**60.5–68.4)	19.9
BMI**									
Underweight/Normal	914	5,622	45.9 (43.3–48.5**)**	46.5 (43.1–49.9**)**	1,186	6,987	51.3 **(**48.3–54.3**)**	50.0 **(**45.1–54.8)	7.6
Overweight	1,081	6,914	49.1 (46.7–51.5**)**	47.6 (44.1–51.2**)**	1,753	10,734	60.4 **(**57.9–62.8**)**	58.9 **(**54.8–62.8)	23.7
Obese	1,387	8,638	61.3 (58.9–63.6**)**	59.6 (56.3–62.7**)**	2,461	14,066	70.1 **(**67.9–72.2**)**	70.7 **(**67.3–73.9)	18.7
**Smoking status**									
Current smoker	655	4,136	48.8 (45.7–51.9**)**	47.9 (44.4–51.4**)**	904	5,451	56.8 **(**53.1–60.5**)**	56.9 **(**52.5–61.2)	18.8
Former smoker	1,170	7,597	52.7 (50.2–55.1**)**	51.6 (47.4–55.8**)**	1,848	10,997	64.1 **(**61.4–66.7**)**	63.6 **(**58.4–68.5)	23.3
Never smoker	1,713	10,418	54.4 (52.4–56.4**)**	53.8 (51.0–56.5**)**	2,845	16,453	61.8 **(**59.8–63.9**)**	62.0 **(**58.2–65.5)	15.2
**Physical activity level**									
Inactive	1,504	8,765	48.4 (46.3–50.5**)**	47.2 (44.0–50.4**)**	2,070	11,485	56.6 **(**54.3–59.0**)**	56.7 **(**52.3–61.0)	20.1
Insufficiently active	762	4,821	57.3 (54.1–60.4**)**	54.2 (49.6–58.7**)**	1,368	8,336	69.2 **(**65.8–72.3**)**	64.7 **(**58.5–70.5)	19.5
Sufficiently active	1,199	8,039	55.3 (53.0–57.6**)**	54.4 (51.6–57.3**)**	2,088	12,608	62.3 **(**60.2–64.4**)**	62.5 **(**59.5–65.3)	14.7
**Have a primary care provider**									
No	261	1,468	42.3 (37.7–47.0)	42.3 (37.6–47.1)	399	2,338	52.9 (46.3–59.4)	50.7 (44.8–56.6)	20.0
Yes	3,292	20,766	53.6 (52.1–55.2)	53.1 (50.9–55.2)	5,190	30,538	62.6 (61.0–64.1)	62.6 (59.9–65.3)	18.0
**No. of annual provider visits**									
None to three	1,075	7,098	46.7 (44.5–49.0)	45.2 (42.4–48.1)	1,999	11,899	56.0 (53.6–58.3)	56.4 (52.8–59.9)	24.7
Four to seven	1,028	6,539	53.3 (50.6–56.0)	55.7 (51.8–59.5)	1,720	10,363	65.1 (62.5–67.6)	63.7 (57.8–69.2)	14.4
Eight or more	1,408	8,373	58.8 (56.6–61.0)	57.8 (54.7–60.9)	1,819	10,311	66.3 (63.8–68.7)	66.1 (62.4–69.6)	14.3
**No. of chronic conditions**									
None	47	276	50.3 (39.4–61.1)	46.0 (34.4–58.1)	111	710	66.2 (57.8–73.6)	63.3 (51.7–73.5)	37.5
One or two	2,089	13,496	51.2 (49.4–53.0)	50.5 (48.3–52.7)	2,993	18,431	58.9 (56.8–60.9)	58.7 (55.8–61.5)	16.2
Three or more	1,436	8,583	55.5 (53.2–57.8)	56.8 (52.3–61.3)	2,535	13,967	65.5 (63.3–67.5)	67.6 (63.2–71.7)	18.9

**Abbreviations:** BMI = body mass index (kg/m^2^); CI = confidence interval.

* Unweighted sample size.

^†^ Weighted number in U.S. population in 1,000s.

^§^ Age-adjusted using the 2000 projected U.S. population.

^¶^ Percentage change calculated using age-adjusted estimates.

** BMI levels: <25.0 underweight/normal weight; 25.0 to <30.0 overweight; ≥30.0 obese.

In both 2002 and 2014, adults with arthritis who did not receive health care provider counseling for exercise had a higher age-adjusted prevalence of physical inactivity. Compared with the referent group of active persons, the prevalence for 2002 was 41.4%, compared with 34.7% (age-adjusted PR = 1.2; CI = 1.1–1.3), and for 2014 was 36.8% compared with 30.5% (age-adjusted PR = 1.2; CI = 1.2–1.3).

## Discussion

Among adults with arthritis, the prevalence of reported health care provider counseling for exercise increased from 51.9% in 2002 to 61.0% in 2014. However, it should be noted that, in a 2014 report, fewer than one third of primary care physicians said they provide exercise counseling for arthritis during office visits (*3*). Although the improvement among all health care providers is encouraging, opportunities exist to further increase counseling for exercise among adults with arthritis. This might be particularly true for some subgroups such as persons who are inactive and who might especially benefit from exercise counseling to help get them started.

Efforts to help health care providers identify patients with arthritis who are inactive, including strategies such as those from Exercise is Medicine (EIM),^¶^ might help facilitate provider counseling for exercise during health care encounters. EIM’s goals are to have clinicians evaluate physical activity levels at every patient visit, assess whether patients are meeting physical activity guidelines, and provide exercise counseling and referral to appropriate therapeutic or community-based physical activity resources. The EIM website has free tools and resources to help providers incorporate these principles to improve chronic disease management in their practices. Other subgroups that have not reached the *Healthy People 2020* target, including underweight/normal weight persons, current smokers, and certain racial/ethnic groups, warrant attention by health care providers during office visits. Adults without a primary health care provider also had a lower prevalence of receiving counseling for exercise. Other health care providers might need to be encouraged to provide exercise counseling, and adults without a primary provider might be encouraged to obtain one.

Health care providers and adults with arthritis agree that physical activity has important benefits for managing arthritis, and federal physical activity guidelines have been found reasonable for adults with arthritis (*3*,*4*). The 2008 Physical Activity Guidelines for Americans** recommend that persons with chronic medical conditions including osteoarthritis, engage in regular physical activity according to their abilities, and highlight that any activity is better than none. Health care providers can serve as valuable sources of exercise advice (*4*), as suggested by the finding that receiving counseling for exercise was associated with lower physical inactivity. However, health care providers often rate their confidence and ability to promote physical activity as low to medium (*5*–*7*). In one study, 61% of health care providers surveyed felt unsure about their knowledge and skills or that they did not have the needed knowledge and skills to provide counseling on exercise to patients with osteoarthritis or rheumatoid arthritis (*8*). Incorporating counseling into clinical training curriculum and continuing education programming (e.g., EIM) might encourage health care providers to provide exercise counseling. Other strategies include incorporating prompts into electronic medical records and training health care providers to provide easily tailored exercise prescriptions.

Providers can reduce arthritis-specific barriers to exercise by referring patients who are uncertain about exercising safely to evidence-based, community programs. Several community group and self-directed exercise programs are available for adults with arthritis (e.g., Enhance Fitness, Walk with Ease, and Active Living Every Day^††^) and can reduce pain and improve function, mobility, and mood.^§§^ Community based organizations, including the National Parks and Recreation Association^¶¶^ and the YMCA*** disseminate these evidence-based physical activity programs throughout the United States.

The findings in this report are subject to at least four limitations. First, NHIS data are self-reported and might be susceptible to recall and social desirability biases. Second, NHIS is only representative of the civilian, noninstitutionalized population, and therefore, estimates do not include those living in long-term care facilities, prisons, or military personnel. Third, low response rates (74.3% in 2002 and 58.9% in 2014) might introduce response bias, although the sampling weights at least partially adjust for this potential bias. Finally, the exercise counseling question does not address the quality or frequency of the counseling.

Prevalence of health care provider counseling for exercise among adults with arthritis has increased significantly over more than a decade, but the prevalence of counseling remains low for a self-managed behavior (exercise) with proven benefits and few risks (*8*), especially among those who are inactive. Various strategies such as health care provider education and training in exercise counseling and electronic medical record prompts might increase health care provider counseling for exercise among adults with arthritis.

**Summary**

**What is already known about this topic?**

The American College of Rheumatology’s osteoarthritis management guidelines recommend exercise as a first-line, nonpharmacologic strategy to manage arthritis symptoms. An estimated 54 million adults in the United States are affected by arthritis.

**What is added by this report?**

The prevalence of receiving health care provider counseling for exercise among adults with arthritis increased 17.6% from 51.9% in 2002 to 61.0% in 2014. However, nearly 40% of adults with arthritis still do not receive health care provider counseling for exercise. In addition, subgroups including non-Hispanic persons of other races, underweight/normal weight persons, current smokers, inactive persons, and persons without a primary health care provider, are still below the *Healthy People 2020* target of 57.4%.

**What are the implications for public health practice?**

Health care provider education and training in exercise counseling, electronic medical record prompts, and connections to community programs might help increase health care provider counseling for exercise among adults with arthritis.

## Footnotes

*
https://www.healthypeople.gov/2020/topics-objectives/topic/Arthritis-Osteoporosis-and-Chronic-Back-Conditions/objectives .

† https://www.cdc.gov/nchs/nhis/quest_data_related_1997_forward.htm.

§
https://www.cdc.gov/nchs/data/statnt/statnt20.pdf .

¶
http://exerciseismedicine.org/ .

**
https://health.gov/paguidelines .

††
https://www.cdc.gov/arthritis/interventions/physical-activity.html .

§§ https://www.cdc.gov/arthritis/marketing-support/compendium/docs/pdf/Compendium-2012.pdf.

¶¶ http://www.nrpa.org/our-work/partnerships/initiatives/healthy-aging-in-parks/.

*** http://www.ymca.net/enhancefitness/.
